# Development of 42 marker panel for in-depth study of cancer associated fibroblast niches in breast cancer using imaging mass cytometry

**DOI:** 10.3389/fimmu.2024.1325191

**Published:** 2024-04-22

**Authors:** Hanna Røgenes, Kenneth Finne, Ingeborg Winge, Lars A. Akslen, Arne Östman, Vladan Milosevic

**Affiliations:** ^1^ Centre for Cancer Biomarkers CCBIO, Department of Clinical Medicine, University of Bergen, Bergen, Norway; ^2^ Department of Pathology, Haukeland University Hospital, Bergen, Norway; ^3^ Department of Oncology and Pathology, Karolinska Institutet, Solna, Sweden

**Keywords:** imaging mass cytometry, tumor microenvironment, cancer associated fibroblasts, microniches, breast cancer

## Abstract

Imaging Mass Cytometry (IMC) is a novel, and formidable high multiplexing imaging method emerging as a promising tool for in-depth studying of tissue architecture and intercellular communications. Several studies have reported various IMC antibody panels mainly focused on studying the immunological landscape of the tumor microenvironment (TME). With this paper, we wanted to address cancer associated fibroblasts (CAFs), a component of the TME very often underrepresented and not emphasized enough in present IMC studies. Therefore, we focused on the development of a comprehensive IMC panel that can be used for a thorough description of the CAF composition of breast cancer TME and for an in-depth study of different CAF niches in relation to both immune and breast cancer cell communication. We established and validated a 42 marker panel using a variety of control tissues and rigorous quantification methods. The final panel contained 6 CAF-associated markers (aSMA, FAP, PDGFRa, PDGFRb, YAP1, pSMAD2). Breast cancer tissues (4 cases of luminal, 5 cases of triple negative breast cancer) and a modified CELESTA pipeline were used to demonstrate the utility of our IMC panel for detailed profiling of different CAF, immune and cancer cell phenotypes.

## 1 Introduction

Imaging Mass Cytometry (IMC) is a formidable method ideal for in-depth study of complex tissue morphology, with the potential to revolutionize histology, histopathology, and diagnostics (1). IMC has been developed based on the earlier available mass cytometry method named CyTOF (cytometry time of flight) known for its capability of detecting c.a. 40 markers in cells in suspension (2–5). High multiplexing of CyTOF is enabled by the implementation of antibodies tagged with various metal isotopes, normally not present in biological tissues, allowing a wider detection range than what is possible using classical IHC techniques. By combining the CyTOF/Helios mass spectrometer system and Hyperion imaging platform, we are able to bring spatial resolution to the high multiplex single-cell proteomic data. This allows to resolve the spatial organization of identified different cell phenotypes, and their distinguished functional states, allowing us to study in depth the tissue architecture and complex communication between cells in health and disease. There are an increasing number of studies based on IMC as the principal method to study tumor microenvironment (TME) in search of novel biomarkers of survival and therapy response (6–10).

Cancer associated fibroblasts (CAFs) represent a heterogeneous population of cells residing in the TME and taking part in building stroma of the solid tumors (11, 12). CAFs are known to be involved in active communication with surrounding cells and to be associated with regulation of tumor immunity and cancer progression (11, 13). Previous studies have demonstrated that CAFs play a major role in controlling tumor biology processes such as epithelial mesenchymal transition (EMT), cancer stemness and proliferation, migration, and metastasis (14–19). In addition, several studies demonstrated the immunomodulatory properties of CAFs, and their ability in orchestrating immunosuppressive effects such as recruitment of T regulatory cells (Tregs), recruitment of myeloid-derived suppressor cells (MDSCs), promotion of M2 phenotype of tumor associated macrophages (TAMs) and negative effect on function of cytotoxic T lymphocytes (CD8+ T cells) (20–28).

Although there are several methodological studies published recently reporting the various antibody panels developed for IMC use (3–5, 29, 30), almost all of these studies (aside from study by 30) are focused primarily on the immune contexture of the TME and largely neglect the mesenchymal component of the TME. In this paper, we are reporting a panel that can be used for a thorough CAF niche-oriented study of breast cancer TME in relation to both immune and breast cancer cell communications.

## 2 Materials and methods

### 2.1 Tissue material

In our study, we relied on archival FFPE tissue collections, obtained from the Department of Pathology at Haukeland University Hospital after acquiring written informed consent from the patients. Tissue samples, before being embedded in paraffin blocks, were fixed in 4% buffered formaldehyde. From the available tissue blocks, we designed two sets of tissue microarrays (TMA). For initial antibody testing and validation, we carefully selected 9 different tissue types as positive controls for our marker candidates (tonsil, lymph node, metastatic lymph node, placenta, adenomatous polyp, and different subtypes of breast cancer (luminal, HER2+, TN, and PDGFRa+ breast cancer) and designed a “test-TMA” consisting of 22 cores in total (**Supplementary Table 1**). The cores were spatially arranged in a manner that there was at least a 5 mm distance between each core, which allowed each “test-TMA” section to be stained with multiple antibodies (antibody per core).

To further examine the utility of our antibody panel in the detection of various subsets of CAFs, in the context of their regulative role in shaping breast cancer immune landscape and governing breast cancer cell properties, we designed a “pilot-TMA”, consisting of 30 breast cancer tissue cores derived from 10 breast cancer patients (4 cases of luminal, 1 case of HER2+ and 5 cases of TN subtype) (**Supplementary Table 2**). Regions of interest were identified by an experienced pathologist with the help of hematoxylin-eosin-stained whole-section slides. The most representative regions of interest to be cored were then carefully selected. TMA blocks were made by punching cores of 1 mm in diameter and mounting them into the recipient paraffin block using a semi-automated precision instrument (Minicore 3, Tissue Arrayer, Alphelys, France). From prepared TME blocks, 4 μm sections were made, mounted on poly-lysine-coated glass slides, and kept at +4°C until further use.

### 2.2 Antibody panel design

In order to be able to detect various aspects of CAF biology and their regulative role in regard to cancer cell functional states and cancer immunity, we carefully selected more than 40 markers we found strongly relevant in obtaining this task (listed in **Table 1**, **Supplementary Table 3**). This set of markers, aside from those targeting fundamental CAF features, included markers specific to breast cancer biology, immune markers, pericyte markers, and endothelial markers. In addition, we included markers to detect specific subcellular compartments useful for cell segmentation (pan-actin, IMC cell segmentation kit – membrane markers, histone H3). To identify the selected set of markers in the tissue, we used the corresponding set of antibodies either commercially available in conjugated form (available from Standard BioTools, San Francisco, CA, USA) or commercially available in a carrier-free formulation, suitable for “in-house “conjugation.

**Table 1:** An overview of antibodies and their corresponding metal isotopes.

Target	Metal tag	Target	Metal tag	Target	Metal tag
CTLA-4	142Nd	aSMA	141Pr	CD68	159Tb
YAP1	144Nd	Vimentin	143Nd	CD8(a)	162Dy
CK 5	149Sm	CD45	145Nd	CD20	164Dy
CD34	151Eu	CD16	146Nd	PD1	165Ho
FAP	152Sm	CD163	147Sm	Ki67	168Er
PDGFRa	160Gd	PanCk	148Nd	CD3	170 Er
pSMAD2	161Dy	PD-L1	150Nd	Cleaved caspase 3	172Yb
ER	163Dy	CD31	151Eu	CK 8/18	174Yb
HER2	166Er	CD44	153Eu	PanAct	175Lu
				Histone H3	176Yb
GATA3	167Er	CD11c	154Sm	IMC segmentation kit 1/3	195Pt
CD24	169Tm	FoxP3	155Gd	IMC segmentation kit 2/3	196Pt
PDGFRb	171Yb	CD4	156Gd	IMC segmentation kit 2/3	198Pt
Granzyme B	173Yb	E-cadherin	158Gd	MCAM	139La
ALDH1	113In	Gamma catenin	115In	pSTAT3	209Bi

Antibodies highlighted in blue were conjugated “in-house” according to the Maxpar X8 conjugation protocol. Antibodies highlighted in yellow were obtained pre-conjugated from the manufacturer (Standard BioTools). Antibodies highlighted in green were conjugated according to Ionpath conjugation protocol. Antibodies highlighted in orange were conjugated according to a modified Maxpar conjugation protocol.

### 2.3 Antibody validation

Conventional chromogenic IHC was used in order to assess antibody performance (sensitivity and specificity) prior to and post-metal-conjugation using whole slide control tissue sections (source of the “test-TMA”). (**Supplementary Figure 1**). Prior to IHC-staining, tissue slides were baked at 60°C for 24h and then deparaffinization, rehydration, antigen retrieval (using cell conditioning solution 1 (CC1, 950-124), pH 9, Roche Diagnostics GmbH, Manheim, Germany), and endogenous peroxidase inhibition (Discovery Inhibitor (760-4840), Roche Diagnostics GmbH, Manheim, Germany) were performed using Ventana Discovery Ultra Platform (Ventana Medical Systems Inc. Tucson, Arizona, USA; Roche Diagnostics GmbH, Manheim, Germany).

After pretreatment in Ventana, slides were collected and washed in warm water with soap to remove the remnants of the liquid coverslip (LCS (650-010), Roche Diagnostics GmbH) and after that in clean warm water to remove the remnants of soap. Followingly, slides were blocked using 3% (W/v) BSA (BSA, A3059-50G, Sigma-Aldrich, Saint-Louis, Missouri, USA) for 45 minutes at RT in a humidity chamber and then incubated with primary antibodies overnight at 4°C. Antibodies were diluted to appropriate concentrations with Dako Antibody Diluent (Antibody Diluent with Background Reducing Components, Dako, Agilent Technologies, Inc. Santa Clara, CA, USA). Following incubation with primary antibodies, tissue sections were rinsed, and followingly washed twice for 5 minutes at RT in a humidity chamber using Dako wash buffer (EnVision FLEX WASH BUFFER 20x, DM831, Dako, Agilent Technologies) and incubated with secondary horseradish peroxidase-conjugated antibodies (Dako EnVision+ System- HRP Labelled Polymer Anti-rabbit; Dako EnVision+ System- HRP Labelled Polymer Anti-mouse, Dako, Agilent Technologies; goat anti-rat (sc-3823), 1:200, Santa Cruz Biotechnology Inc., Santa Cruz, CA) for 30 minutes at RT in a humidity chamber. Following incubation with the secondary antibodies, slides were washed twice for 5 minutes using Dako washing solution (EnVision FLEX WASH BUFFER 20x, DM831, Dako, Agilent Technologies) in a humidity chamber at RT. The staining patterns were revealed using diaminobenzidine chromogenic substrate (DAB, Dako Liquid DAB+ Substrate Chromogen System, K3468 Dako, Agilent Technologies), after incubation for 10 minutes in a humidity chamber at RT. Tissue sections were then washed with distilled water and counterstained with hematoxylin (Dako Automation Hematoxylin Histological Staining Reagent, S3301, Dako, Agilent Technologies) for 10 minutes, after which they were washed for 5 min using tap water. Following this, tissue sections were dehydrated with increasing concentrations of ethanol and xylene and then mounted using an automated mounting machine (CoverSliper CR100, Dako-Agilent, Copenhagen, Denmark).

Assessment of staining quality for each individual antibody was performed using a Leica microscope (DM2000 LED, Leica Microsystems CMS GmbH, Wetzlar, Germany). Antibodies that performed satisfactorily were selected, and the optimal concentrations to be used for further analysis were assessed.

### 2.4 Antibody metal conjugation

Of the 43 antibodies, 26 antibodies were purchased in an already conjugated form from Standard BioTools, and the remaining 17 antibodies were conjugated “in-house” (**Table 1**). From the 17 antibodies that were conjugated “in-house”, 13 antibodies were conjugated to lanthanide metal isotopes available in the Maxpar antibody labeling kit (Maxpar^®^ X8 Multimetal Labeling Kit—40 Rxn,Standard BioTools)). Anti-pSTAT3 antibody (clone D3A7, Cell Signaling Technology, Danvers, MA, USA) was conjugated to 209Bi according to a modified Maxpar Conjugation protocol by Han et al. (2), using high-purity bismuth III-nitrate pentahydrate salt (254150-25G, Sigma-Aldrich, Saint-Louis, Missouri, USA). Anti-MCAM antibody (clone HPA008848, Atlas Antibodies, Bromma, Sweden) was conjugated to 139La according to the modified Maxpar Conjugation protocol by Elaldi et al. (5), using high-purity lanthanum (III) chloride heptahydrate salt (203521-25G, Sigma-Aldrich). ALDH1 and Gamma-catenin were conjugated with 113In and 115In, respectively, using the Ionpath conjugation protocol and reagents (Ionpath, Menlo Park, CA, USA). Additionally, we conjugated a goat anti-rabbit IgG secondary antibody (A16098, Invitrogen, Waltham, Massachusetts, USA) to 160Gd using Maxpar X8 conjugation protocol and reagents. After conjugation, protein concentrations were measured using Nanodrop (ND-1000, NanoDrop, Spectrophotometer, Saveen Werner, Malmö, Sweden) to ensure that a sufficient amount of antibody was retrieved. Following conjugation, all antibodies after elution were further diluted in 20 µl of Antibody Stabilizer PBS (Candor Bioscience GmbH, Wangen, Germany) and stored at +4°C until further use.

In order to exclude the possibility that the conjugation process substantially affected the performance of the antibodies, the validated antibody panel was tested by conventional IHC as previously described and immunodetection patterns were compared to their non-conjugated counterparts before performing IMC staining.

### 2.5 Antibody panel validation and titration using IMC

Prior to IMC-staining, TMA-slides were baked for 2h at 60°C and preprocessed in Ventana using a similar protocol to one used for IHC staining, omitting in this case the endogenous peroxidase inhibition. After pretreatment in Ventana, slides were collected and washed in warm water with soap to remove the remnants of the liquid coverslip and again in clean warm water to remove the remnants of soap). Followingly, slides were blocked using 3% (W/v) BSA) for 45 minutes at RT in a humidity chamber. Slides were first incubated with primary nonconjugated anti-PDGFRa diluted to 1:100 for 1h at RT, after which the slides were washed twice for a duration of 8 min in a Coplin jar with freshly prepared 0.2% Triton x-100 (Sigma-Aldrich) PBS solution and then again for 8 min with MilliQ water on an orbital shaker plate (130 Basic, IKA KS, Staufen, Germany) with gentle agitation (160 rpm). Following incubation with anti-PDGFRa, slides were incubated for 30 min at RT with 160Gd conjugated secondary anti-rabbit antibody in three concentrations (1:400, 1:800, and 1:1200). After incubation with the secondary antibody slides were washed in the same manner as after incubation with the primary antibody. Cocktails of primary antibodies were prepared at three different consecutive concentrations (each primary antibody was used in three consecutive serial dilutions, with each subsequent dilution being half the concentration of the preceding)and slides were incubated overnight at +4°C in a humidity chamber. All antibodies were diluted to given concentrations with Dako Antibody Diluent (Antibody Diluent with Background Reducing Components, Dako, Agilent Technologies). After incubation with antibody cocktails, slides were washed as described above and stained with Iridium Intercalator (Standard BioTools), diluted previously 1:4000 in PBS, and incubated for 30 min at RT. Subsequently, slides were washed with MilliQ water for 5 minutes on an orbital shaker and then dried at RT for at least 20 min before acquisition in the Hyperion mass cytometry system.

Prior to the acquisition, the Hyperion mass cytometry system was autotuned using a 3-element tuning slide (Standard BioTools) according to the tuning protocol provided by the Hyperion imaging system user guide (Standard BioTools). The inner quadrants of the cores are being acquired and data were exported as MCD files and visualized using the MCD™ viewer (Standard BioTools). Appropriate dilution ratios for each of the antibodies in the panel were assessed visually. After the assessment of adequate dilution ratios for each antibody in the previous step, the complete master mix containing 43 antibodies was applied to “test-TMA” in IMC-staining for further evaluation.

### 2.6 Quantitative and qualitative evaluation of IMC-staining

For this purpose, staining data for each individual antibody was collected from consecutive, or close to consecutive (up to 4 sections distance), “test-TMA” sections using both IHC and IMC as described above. IHC staining was performed on 3 slides in total, where each single antibody was used to stain the single TMA core (**Supplementary Table 1**). The stained slides were then scanned using NanoZoomer-XR (Hamamatsu Photonics K.K., Shizuoka, Japan) using a 40x objective. Aperio ImageScope 12.4.3.5008 software was used to visualize and extract.tiff files from each core. Raw IMC data were visualized using MCD™ viewer (Standard BioTools) and 8-bit two-channel .tiff files were extracted showing the marker of interest and nuclear staining. Obtained IMC data showing individual marker staining patterns were then compared visually, side-by-side, to IHC staining patterns taken as a ground true for qualitative evaluation. Furthermore, quantitative evaluation was performed in the form of IMC/IHC correlation analysis of consecutive sections. For this purpose, QuPath (31) was used to retrieve data for further correlation analysis. Within the software,for each marker, both the IMC and IHC images were segmented into tiles, giving 25 tiles per image (**Supplementary Figures 2A, B**). Each tile within the image was compared to the corresponding tile in the IHC image. For the 25 respective tiles, both the mean signal intensity measure and positive cells detected for each marker were counted (**Supplementary Figure 2C**). To detect cells positive for specific markers, cell detection was first performed in QuPath based on nuclear staining and then specific marker positive cells were detected using single measurement classifier. On the basis of acquired data, Spearman correlation analysis was performed to examine the correlation between the measurements, and Bland-Altman plots were designed to challenge the concordance between the two methods.

### 2.7 Pilot-TMA preparation, IMC data acquisition and data preprocessing

Following panel validation and correlation analysis of “test-TMA”, the panel has been further examined for its capacity to detect the biological contextures of breast cancer TME. For this purpose, the “pilot-TMA” was stained, and IMC data was acquired as described above.

Obtained raw IMC data was first transformed into .tiff files, preprocessed, and segmented into individual cells (pan-actin and IMC cell segmentation kit were used as cytoplasm/membrane markers and histone H3 and iridium intercalator staining were used as nuclear markers) using the Steinbock framework (version 0.15.0) pipeline according to Windhager et al. (32) (details are available at https://bodenmillergroup.github.io/steinbock/latest/). Obtained .tiff images have been corrected for channel crosstalk (**Supplementary Figure 3**) as described in work by Chevrier et al. (33). Extracted single-cell intensity data were then used with the modified CELESTA pipeline (34) to detect biologically meaningful cell phenotypes.

### 2.8 Cell classification

We used a modified approach in running the CELESTA algorithm in the terms that we performed stepwise cell classification using first the so-called “first level markers” (PanKeratin, CD31/CD34, vimentin, E-cadherin, CD45 and MCAM) to detect 5 main cell classes (epithelium, immune cells, fibroblasts, endothelial cells and pericytes). After defining the main classes, different subclasses of fibroblasts, immune cells and epithelial cells were further assessed by running the CELESTA algorithm separately for each class, which enabled detection of high-resolution cell subsets and better control over the data [**Supplementary Figures 4**-**7**, (detailed information and data sample is available at https://osf.io/eu8ct)].

All outputs from different runs were combined using a Python script. The script followed the logic where in the case that a cell has been classified differently in different steps (e.g. a cell classified as epithelial cell in the first run and then as a CD4+ T cell in the immune cell run) the cell was then classified as “ambiguous”. Due to the marker combinations used for the identification of immune cells (CD45+, vimentin+/-) and fibroblasts (vimentin+), we allowed cells classified in some of the steps as “fibroblasts” not expressing other fibroblast markers used for subclassification (e.g. aSMA, FAP, PDGFRa, PDGFRb, Yap1 and pSMAD2), but expressing immune specific markers used for subclassification (e.g. CD3, CD20, CD68, CD4, CD8, FoxP3, GranB, PD1, CD11c, CD163, CD16 and CTLA4) to be named as corresponding immune cell subclass. Due to the expression of MCAM in some of the tumor cells, the logic also allowed cells that are classified as “pericytes” and “tumor cells” to be labeled as “tumor cells”. In the case where a cell has been classified as “unknown” in all the run rounds, due to the inability to resolve its identity based on marker combination and spatial orientation in regard to other cells, that cell would remain labeled as “unknown”. Cell phenotypes that ended in having only one representative across the dataset were considered potential artifacts and reclassified as “unknown” cell class. This resulted in two additional main cell classes in the output: “unknown” and “ambiguous”.

### 2.9 Statistical analysis

We used Spearman two-tailed test to determine the correlation between continuous variables, with Spearman correlation coefficient values higher/lower than ± 0.5 and p-values < 0.05 regarded as significant. Spearman correlation test was performed, and Bland-Altman plots were constructed using SPSS version 26 (SPSS Inc., Chicago, IL). We used the R script and ᵡ2 test to examine the inter and intracase heterogeneity and variations in cellular compositions between luminal and triple negative breast cancer (p-values < 0.05 were considered significant). For the assessment and graphical representation of various cell class distributions, we used Python and R scripts.

## 3 Results

### 3.1 IMC panel demonstrated high specificity and sensitivity when tested on control tissues

Our antibody candidates underwent a strict quality control process where we assessed the specificity and sensitivity of each antibody prior to conjugation using the IHC method (**Supplementary Figure 1**). All antibodies have been tested in IHC after conjugation (including the antibodies obtained from Standard BioTools) in order to ensure that they retained their affinity after the conjugation process. We have observed that in the case of the anti-PDGFRa antibody (clone D13C6, Cell Signaling Technology), the signal was not present in IHC after conjugation (**Supplementary Figures 1G, H**) indicating that the conjugation process altered the function of this antibody. As anti-PDGFRa showed a significant sensitivity to conjugation, and as this antibody was crucial for our CAF-focused panel, to bypass this obstacle, we included a secondary anti-rabbit antibody (A16098, Invitrogen, Waltham, Massachusetts, USA) in our panel to be used together with unconjugated anti-PDGFRa.

Following the conjugation step, the IMC panel was tested using “test-TMA”, where we compared the quality and patterns of IMC staining of each of the antibodies to the IHC as a ground truth. With this, we wanted to ensure that the metal conjugation was performed successfully and that the labeling of the antibodies did not interfere with their performance.

**Figure 1:**
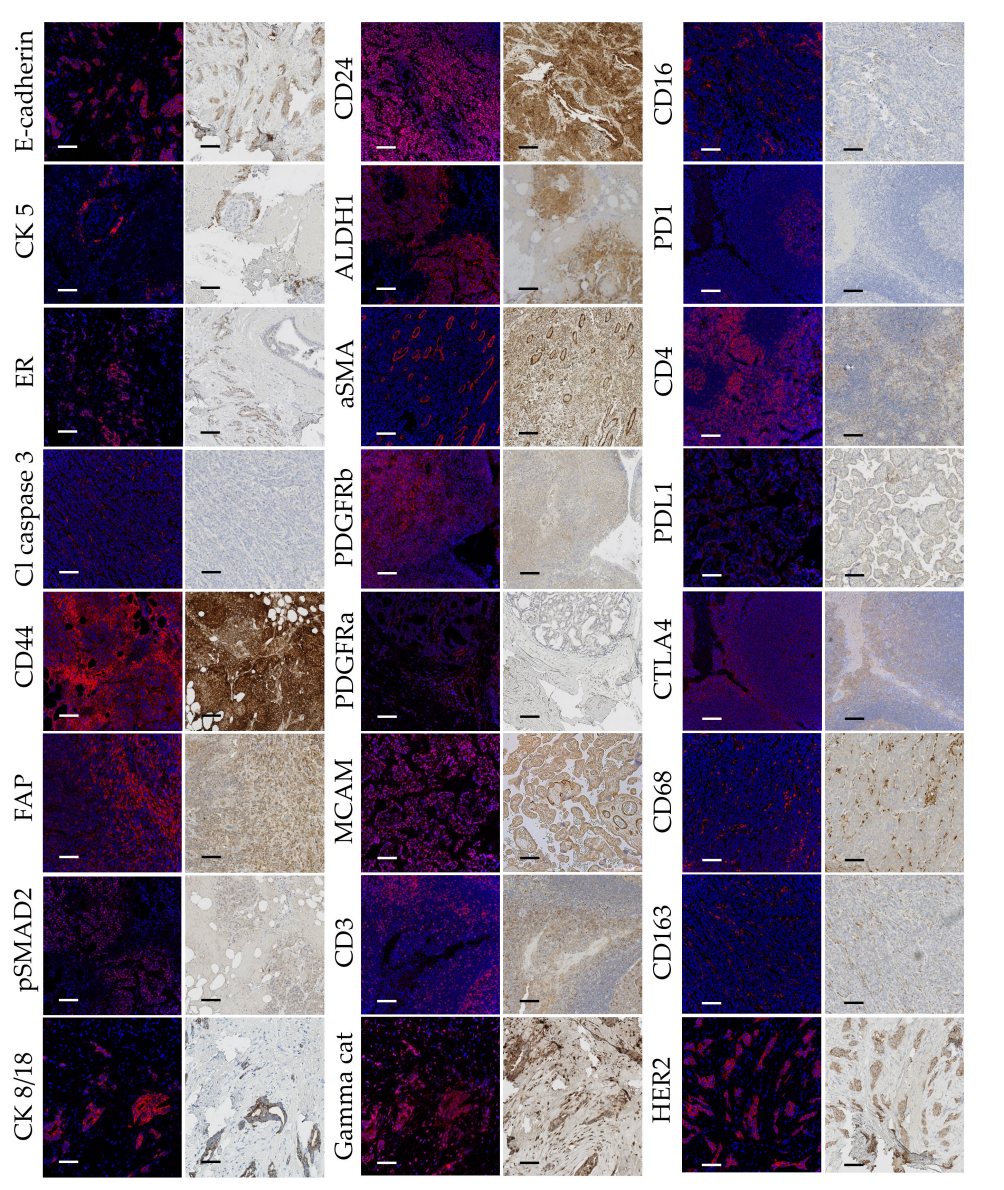
Comparative side by side representation of IMC and IHC staining patterns. Image pairs consist of IMC-acquired images presented to the left, and IHC micrographs presented to the right, both obtained from the same cores of the “test-TMA”. Positive antibody staining is visualized with red in the IMC images, and brown (DAB) in the IHC images. Blue color corresponds to nuclear staining. Cl Caspase 3, Cleaved Caspase 3; PanCK, Pan Cytokeratin. Each scale bar corresponds to 100 µm.

After visual inspection of each channel to assess the overall signal quality, we performed qualitative panel evaluation by visually controlling each channel and comparing the staining patterns with IHC staining (**Figure 1**). All antibodies showed satisfactory staining and comparable staining patterns except anti-pSTAT3 (clone D3A7, Cell Signaling Technology, 209Bi labeled). Conjugated anti-pSTAT3 showed acceptable staining quality in IHC (**Supplementary Figure 1F**) and the IMC signal was transiently present for this antibody after the conjugation. Nevertheless, after the period of 12 months after conjugation, there was no signal detected in IMC indicating poor stability of this type of conjugate.

**Figure 2:**
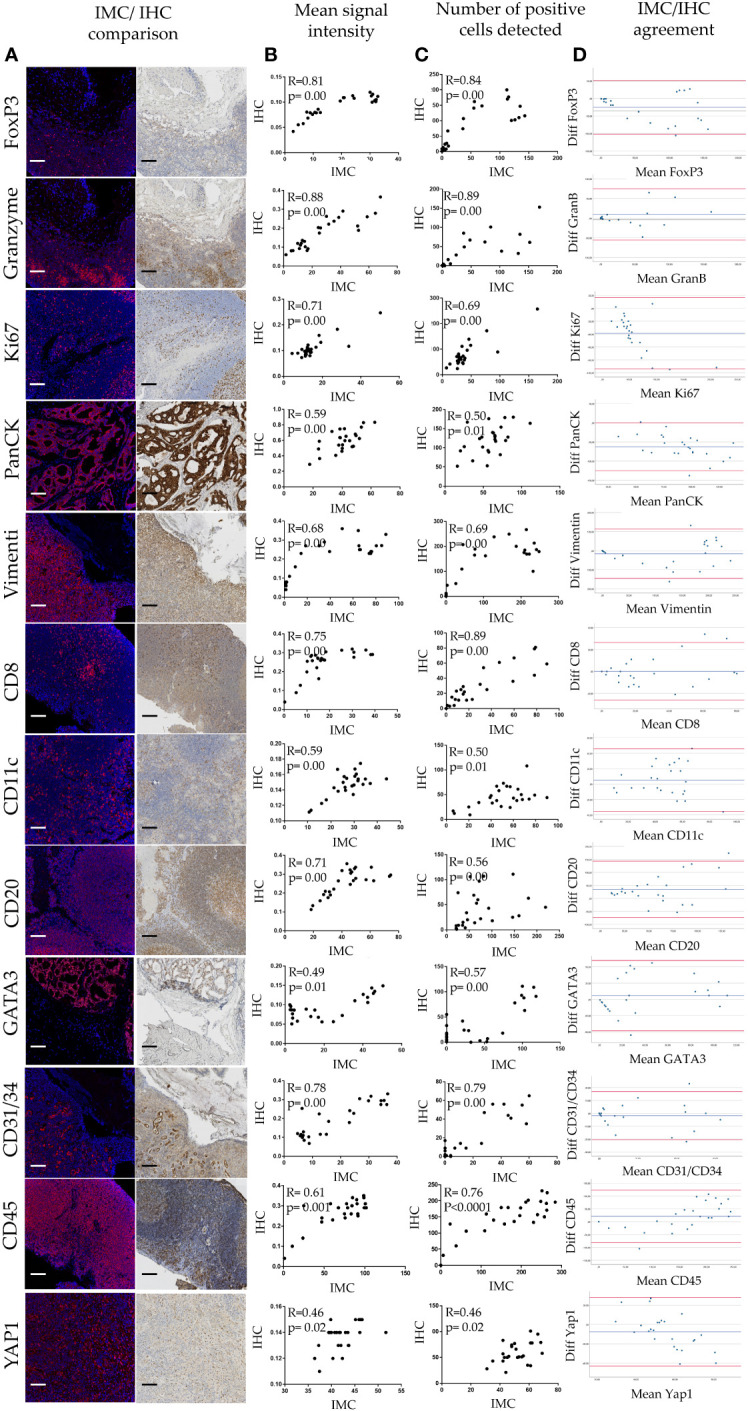
Quantitative analysis of IMC staining. **(A)** IHC/IMC side-by-side image representation. Each scale bar corresponds to 100 µm. **(B)** Scatterplots showing associations of mean signal intensity between IHC and IMC staining for given antibody. **(C)** Scatterplots showing associations of positive cell detection between IHC and IMC staining for given marker. **(D)** Bland-Altman plots showing IMC/IHC concordance in detection of cells positive for a given marker.

**Figure 3:**
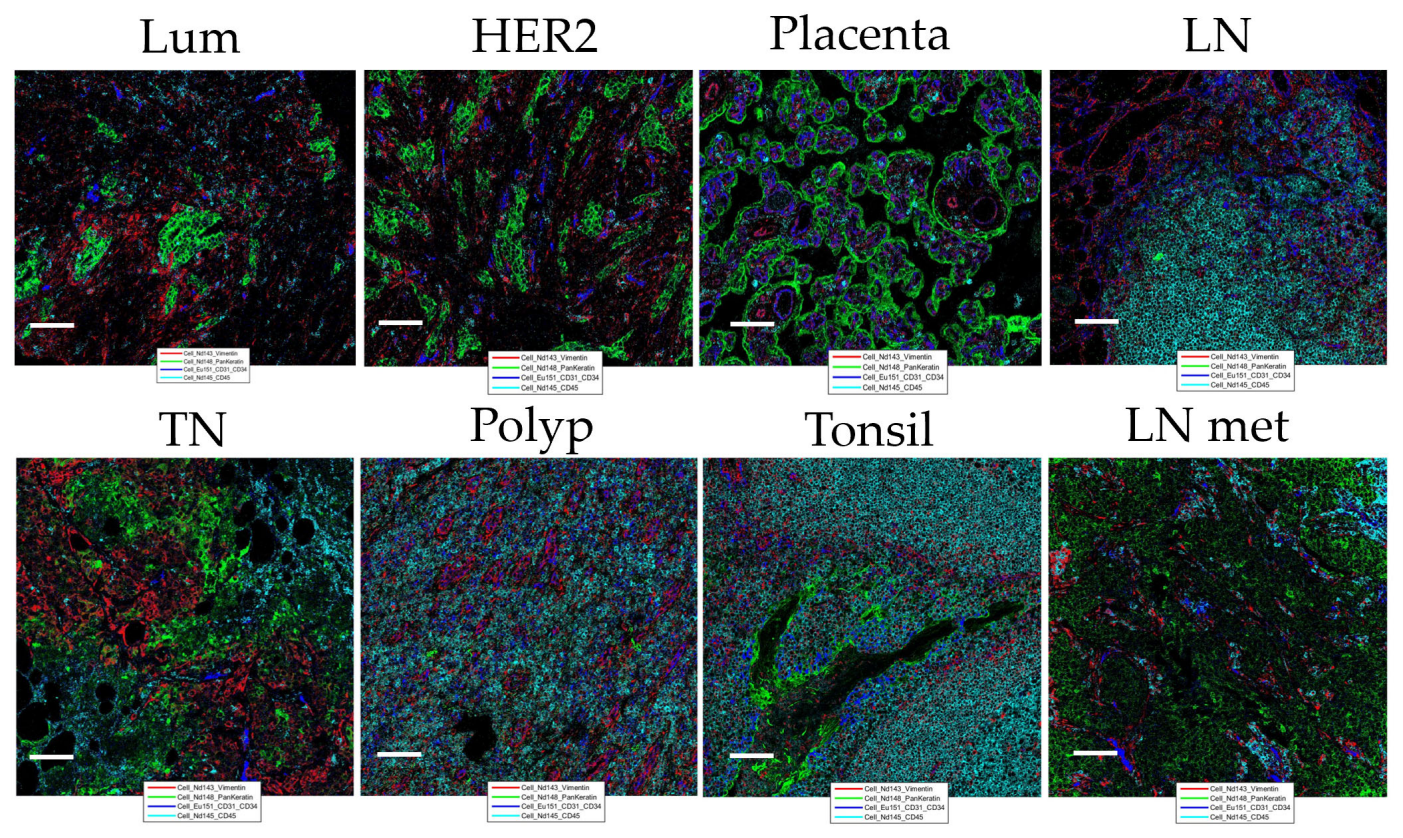
Representation of “test-TMA” IMC staining Images show staining patterns of the level 1 classification markers: Vimentin (red), PanKeratin (PanCK) (green), CD31/34 (blue), and CD45 (turquoise). Lum, Luminal; TN, Triple-negative and HER2, HER2-enriched. Each scale bar corresponds to 100 µm. LN, lymph node; LN met, metastatic lymph node.

Quantitative evaluation of IMC staining was performed on the consecutive “test-TMA” sections where staining intensity and number of marker positive cells coming from IHC and IMC data were correlated. Spearman correlation coefficient showed an overall strong correlation between IHC and IMC staining when compared for the staining intensity and number of positively detected cells per image area (tile) (**Figure 2**), with the highest correlation coefficients observed for granzyme B with a Spearman correlation coefficient value of 0.88 for signal intensity and 0.89 for the number of positive cells detected. Although we noted an overall high correlation between IHC and IMC staining, we observed exceptions in the case of CD24 and CD163 (**Supplementary Table 4**). These variations do not necessarily indicate poor reliability of the antibodies and methods used, as the opposite has been demonstrated in **Figure 1**, but is most likely caused by variability in the tissue morphology of different sections used for staining, and/or possibly due to a larger dynamic range of the signal displayed in IMC data. Following Spearman correlation analysis, Bland-Altman plots were constructed to further evaluate the concordance between the two methods. We demonstrated high concordance, regarding positive cell detection in IMC, when compared to IHC as a ground truth (**Figure 2D**). Prior to testing the panel in the “pilot-TMA” setting, the quality of the IMC staining has been assessed one more time in the “test-TMA” where visually, each channel has been controlled for signal quality and staining specificity in each of the available control tissues (**Figure 3**).

**Figure 4:**
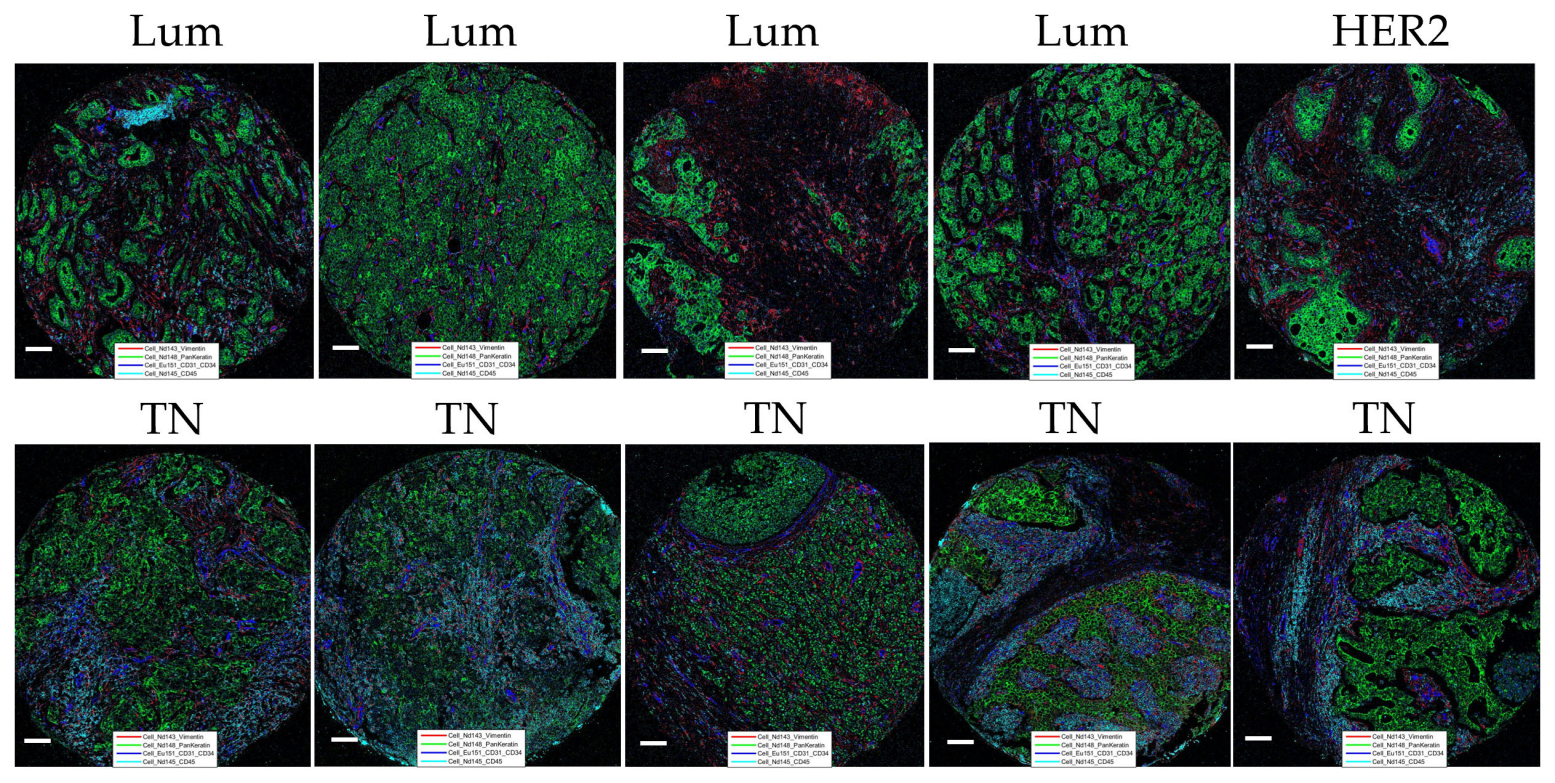
Representation of “pilot-TMA” IMC staining. Images show staining patterns of the level 1 classification markers: Vimentin (red), PanKeratin (PanCK) (green), CD31/34 (blue), and CD45 (turquoise). Lum, Luminal; TN, Triple-negative and HER2, HER2-enriched. Each scale bar corresponds to 100 µm.

Following thorough IMC panel validation in the “test-TMA” setting, where it has been concluded that the panel has displayed overall good quality and concordance with the IHC staining, the complete panel was used for staining of “pilot-TMA”, consisting of five cases of Luminal, four cases of triple negative, and one case of HER2 enriched breast cancer subtypes (each case represented by multiple numbers of cores) (**Supplementary Table 2**). Following a thorough visual examination of every channel within the available cores, ensuring optimal staining quality (**Figure 4**), the “pilot-TMA” imaging data underwent further analysis to confirm the relevance of the panel in characterizing distinct cell subclasses.

### 3.2 IMC panel demonstrated substantial capability in resolving different cell subtypes in high resolution and displayed strong potential for studying CAF-regulated microniches

The focus of this study is to only report on the development of the IMC panel for in-depth study of CAF controlled micro niches in breast cancer tumor microenvironment. As the cell classification approach used in this study requires further refinement and the number of used breast cancer tissue samples is low, presented data describing various identified cell classes serves only to support and demonstrate the panel’s potential and is not intended to describe any conclusive biological findings.

**Figure 5:**
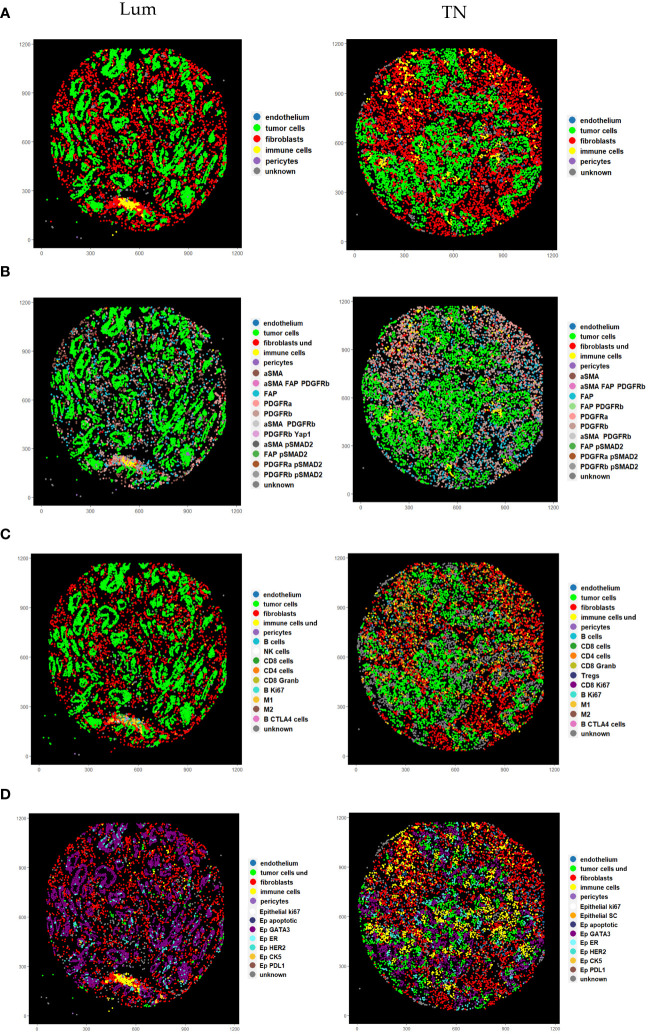
Pseudo images showing cell phenotypes identified using stepwise CELESTA approach. **(A)** Representation of the main cell classes identified in the first step. **(B)** Representation of different CAF phenotypes identified in the second step. Label “fibroblasts und” corresponds to CAFs with undefined phenotypes **(C)** Representation of different immune phenotypes identified in the second step. Label “immune cells und” corresponds to immune cells with undefined phenotypes **(D)** Representation of different cancer phenotypes identified in the second step. Label “tumor cells und” corresponds to tumor cells with undefined phenotypes. Lum, Luminal and TN, Triple-negative.

In order to demonstrate the utility of our panel in resolving the CAF cell subtypes in high resolution, we focused on luminal (Lum) and triple negative (TN) breast cancer tissue from “pilot-TMA”. After performing cell segmentation, we identified 126 657 cells in total, of which 57 662 cells were detected in Lum breast cancer tissue and 68 995 cells in TN breast cancer tissue (**Supplementary Table 5**). These cells were subjected to CELESTA cell classification pipeline in order to differentiate between different cellular phenotypes. We used a stepwise approach, where we first defined main cell classes (immune cells, fibroblasts, pericytes, tumor cells and endothelial cells) from the total bulk of cells from each core and then individually split fibroblasts, epithelial and immune cells into corresponding cell subclasses (**Figure 5**) using relevant marker combinations (**Supplementary Figures 4**-**8**; see “Material and Methods” section). Among these five main classes, we as well identified cells labeled as “unknown” (cells whose identity couldn’t be resolved using our panel and presented classification approach) and a cell population labeled as “ambiguous” (cells showing biologically unlikely marker combinations).

**Figure 6:**
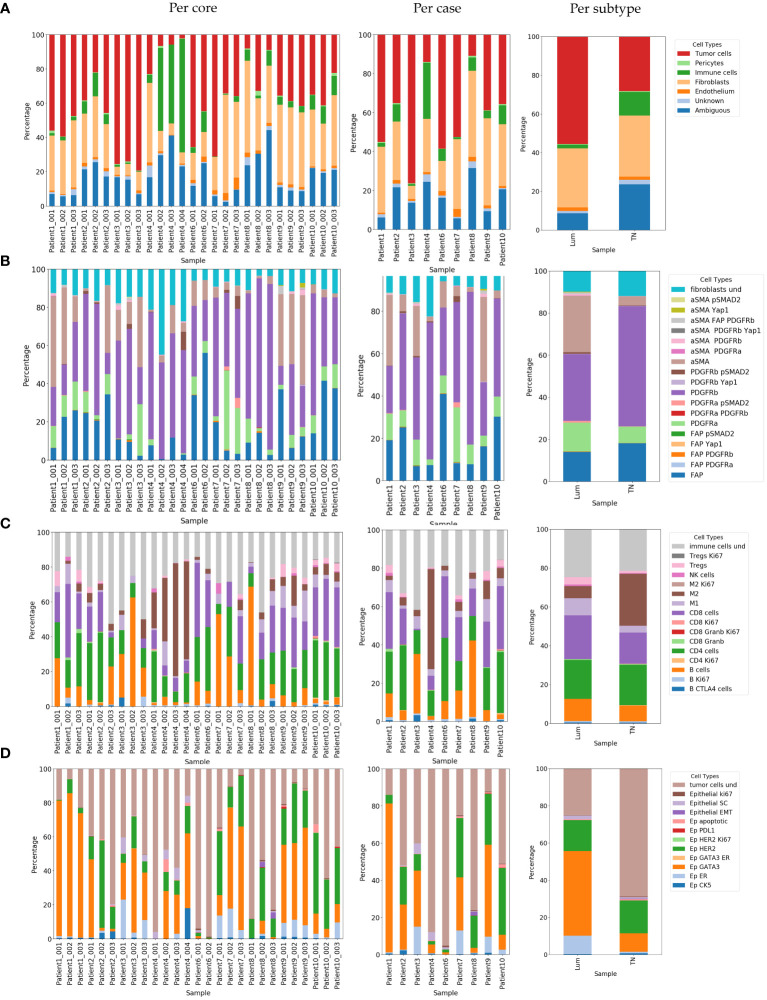
Relative abundance of identified cell phenotypes demonstrated per core, per case and per subtype. **(A)** Relative abundance of identified main cell classes. **(B)** Relative abundance of identified CAF phenotypes. Label “fibroblasts und” corresponds to CAFs with undefined phenotypes **(C)** Relative abundance of identified immune phenotypes. Label “immune cells und” corresponds to immune cells with undefined phenotypes **(D)** Representation of identified cancer phenotypes. Label “tumor cells und” corresponds to tumor cells with undefined phenotypes. Lum, Luminal and TN, Triple-negative.

We observed intra (shown as cell abundance per core in **Figure 6A**) and inter-case variability (shown as cell abundance per case in **Figure 6A**) in cellular composition. When examining the variability of the main cell classes, in the majority of cases, intra-case variability was not statistically significant (Patient 1, Patient 2, Patient3, Patient 9 and Patient 10). Nevertheless, in cases Patient 4 Patient 6, Patient 7 and Patient 8 we did discover statistically significant intra-case heterogeneity in main class cellular composition (**Supplementary Table 6**). This was more common in TN breast cancer cases (Patient 4, Patient 6 and Patient 8), indicating high heterogeneity in this tumor subtype. Although we observed a certain degree of intra-case variability, inter-case variability was generally greater (ᵡ2 = 253.7; df=48; p< 2.2e-16).

There was a noticeable difference in the cell composition of some of the main cell types between the two breast cancer subtypes. The portion of the “ambiguous” cell population was greater and statistically significant in favor of TN breast cancer (ᵡ2 = 6.9; df=1; p=0.009) when it was generally low in the luminal breast cancer subtype. We also observed a significant difference in the portion of immune cell populations in favor of TN breast cancer (ᵡ2 = 7.5; df=1; p=0.006) and cancer cell populations in favor of Lum breast cancer (ᵡ2 = 8.96; df=1; p=0.003) (shown as cell abundance per subtype in **Figure 6A**).

Using our panel and adapted CELESTA approach, we were able to resolve in total 19 distinct CAF populations (**Figures 5B** and **6B**, **Supplementary Table 7**). Comparing the abundance of these CAF populations, we observed high intra-case and inter-case heterogeneity in CAF composition (shown as cell abundance per-core and per-case in **Figure 6B**, **Supplementary Table 6**). We observed a significant intra-case variability in the majority of the cases, except in Patient 6 and Patient 10 (**Supplementary Table 6**). Nevertheless, the inter-case heterogeneity (ᵡ2 = 332.01; df=144; p<2.2e-16) was greater.

Continuing analysis in breast cancer subtypes, we observed different CAF cell populations occurring in Lum and TN breast cancer (**Figures 5B** and **6B**; **Supplementary Table 7**, **Supplementary Figure 9**). Overall, Lum breast cancer had higher percentage of aSMA expressing (statistically significant, ᵡ2 = 16.14; df=1; p=5.9e-05) and PDGFRa (statistically not significant, ᵡ2 = 1.59; df=1; p=0.21) expressing CAFs (**Figures 5B** and **6B**; **Supplementary Table 8**) in contrast with TN breast cancer which had significantly higher percentage of PDGFRb+ (ᵡ2 = 7.27; df=1; p=0.007) and slightly higher percentage of FAP+ CAFs (statistically not significant, ᵡ2 = 0.51; df=1; p=0.47) when compared to Lum (**Figures 5B** and **6B**; **Supplementary Table 7**). When comparing the abundance of immune subclasses between cases and cores, we observed overall high inter-case and intra-case heterogeneity (shown as cell abundance per core and per case in **Figure 6C**). Although there was a significant intra-case variability in cellular composition in majority of the cases, except in Patient 6 and Patient 10 (**Supplementary Table 6**), the observed inter-case heterogeneity was significantly higher (ᵡ2 = 484.77; df=120; p<2.2e-16).

Looking at these cells in more detail, we detected in total 16 different immune cell populations (**Supplementary Table 8**, **Figure 6C**). The occurrence of these cells was different between Lum and TN breast cancer both from the aspects of presence and abundance. In TN breast cancer we identified proliferating CD4 and CD8 T cells (**Figures 5C** and **6C**; **Supplementary Table 8**). When looking into main immune classes, Lum breast cancer showed higher percentage of B and T cells, and NK cells when compared to TN breast cancer (non-statistically significant; B cells, ᵡ2 = 0.55; df=1; p=0.46; T cells, ᵡ2 = 0.72; df=1; p=0.40; NK cells, ᵡ2 = 0.27; df=1; p=0.60), which had significantly higher percentage of macrophages in comparison with luminal breast cancer (ᵡ2 = 5.1; df=1; p=0.024) (**Figures 5C** and **6C**; **Supplementary Table 8**, **Supplementary Figure 10A**). Of all macrophage populations the highest portion in TN breast cancer was M2 population with 88.29% of all macrophages detected in this tissue type (ᵡ2 = 16.3; df=1; p=5.4e-05) (**Supplementary Figure 10D**; **Supplementary Table 8**) comparing with Lum that had a higher portion of M1 macrophage population with 57.83% (ᵡ2 = 31.01; df=1; p=2.56e-08) (**Supplementary Figure 10D**, **Supplementary Table 8**). Although there was a certain variety in cellular abundance, there was no statistically significant difference detected in the composition of different subclasses of T and B cells between breast cancer subtypes (**Figures 5C** and **6C**; **Supplementary Table 8**, **Supplementary Figure 10A**). In patient 1 (Lum), Patient 6 (TN) and Patient 8 (TN) we detected tertiary lymphoid structures (TLS) structures (**Supplementary Figure 11**).

Looking into identified epithelial cell populations, we observed overall high inter-case and intra-case heterogeneity (shown as cell abundance per core and per case in **Figure 6D**).With exception of the cases: Patient 1, Patient 6 and Patient 9, there was overall a significant intra-case variability in cellular composition (**Supplementary Table 6**) Although we observed high intra-case heterogeneity, generally the inter-case heterogeneity was greater (ᵡ2 = 569.5; df=88; p<2.2e-16).

Expectingly, Lum breast cancer showed a higher percentage of detected estrogen receptor (ER) and GATA3 positive cells (ᵡ2 = 7.5; df=1; p=0.0062 and ᵡ2 = 22.79; df=1; p=1.8e-06, respectively) (**Figures 5D** and **6D**; **Supplementary Table 9**). TN breast cancer tissues showed a higher percentage of CK5 expressing epithelial cells (non-statistically significant, ᵡ2 = 0.08; df=1; p=0.78) (**Figures 5D** and **6D**; **Supplementary Table 9**). TN breast cancer tissues also had higher portions of proliferating cancer cells, cancer cells undergoing EMT, apoptotic cells, and lower percentage of cancer stem cells, although not statistically significant (**Figures 5D**, **6D**; **Supplementary Table 9**).

## 4 Discussion

In this study we reported a 42 marker IMC panel suitable to be used on human FFPE tissues and suggested a supervised cell classification approach that together have a strong capacity of detecting high-resolution CAF populations, as well as in depth profiling of immune and cancer cell populations. This altogether allows in-depth studying of biological contexture of CAF microniches in the focus of tumor microenvironment with an aim of detecting novel markers of prognosis of invasive breast cancer and beyond.

As a first step of designing our panel, we put an effort in carefully examining each antibody in order to ensure that they perform satisfactory in the term of sensitivity and specificity before and after conjugation process, as being strongly suggested in work by Ijsselsteijn et al. (3). To evaluate the performance of our antibodies, we used IHC staining as a ground truth to compare the intensity and specificity of the signal obtained using IMC. To minimize the number of sections needed for IHC staining and assure consecutive sections for analysis, we designed “test TMA” block in a way that allowed staining with multiple antibodies per section (staining per core), which allowed us to have staining data from consecutive sections and more precise comparison between IHC and IMC staining. We put an effort in expanding the spectrum of usable channels in IMC by conjugation of antibodies with metals not available in the conventional Maxpar conjugation kit (In, La and Bi). We conjugated anti-MCAM and anti-pSTAT3 antibody “in-house” as reported in work by Han et al. (2, 35) and Elaldi et al. (5). Although both conjugated -anti MCAM and anti-pSTAT3 antibody performed well initially after conjugation, after the period of around a year, anti-pSTAT3 antibody didn’t give any signal in IMC. One possible explanation is that bismuth metal conjugates are less stable, and polymer used in Maxpar conjugation is not suitable for chelation of non-lanthanide metals. This is in contrary to study by Han et al., (35) where they reported successful conjugation with BiNo3 salts using Maxpar reagents and reported that the conjugated antibody was stable for up to two years following conjugation. We also detected issues when trying to conjugate anti-PDGFRa antibody using Maxpar reagents with Gd metal isotope 160. It has been already reported that some antibodies are more sensitive to conjugation process, particularly to the reduction step, which has for aim to brake disulfide bonds and allow polymer to covalently bind to the antibody (3). Our anti-PDGFRa happened to be particularly sensitive to the conjugation process and has been shown to lose its function after conjugation. As this antibody was of a strong interest to us, we bypassed this problem by introducing conjugated secondary anti-rabbit antibody, which was used in hybridization together with primary non conjugated anti PDGFRa to detect its signal in IMC.

In order to evaluate the antibody performance, we undertook quantitative and qualitative analysis of IHC and IMC staining, where we first visually assessed the staining quality and following this, we performed a quantitative analysis. Our quantitative analysis was based on using consecutive tissues sections, where by using a specific design of our “test-TMA” (distance between each core was at least 5 mm), we were able to perform staining with different antibodies per each core using the same section. This helped us to have all the antibodies tested and staining performed on only 4 different sections, and therefore enabled us to perform as precise a comparison of the staining as possible. We correlated staining intensity but also the number of detected cells for each marker. Overall, our quantification approach showed that most of the antibodies we used in our panel correlated strongly with the staining we obtained in IMC when compared to corresponding single antibody staining in IHC. We do acknowledge that the correlation coefficient was lower for certain antibodies, which has been evidently caused by the differences in tissue morphology present in different tissue sections, as clearly demonstrated when staining patters are compared side by side.

We also analyzed concordance in number of cells identified as positive for individual markers using IHC staining as a ground truth, as described previously in study by Jackson et al. (6), and we concluded high reliability of signal coming from IMC in detection of truly positive cells for markers used.

Previous studies based on IMC, usually focused on different clustering approaches for cell phenotypization (6, 32, 36–40). In our cell classification approach, we used CELESTA (34) to first identify five main classes expected to be present in breast cancer tissue (cancer cells, immune cells, vessels, pericytes and CAFs) which were than further subclassified. Alongside the main cell classes, we as well identified population of cells being labeled as “unknown”, meaning that their identity couldn’t have been resolved based on the markers and data analysis parameters we used. The portion of these “unknown” cells varied between the cores and cases, but overall, in both breast cancer subtypes this population accounted for a very small percentage, indicating the precision and robustness of our panel and cell classification approach. With implementing the stepwise approach in cell classification, we as well identified so called “ambiguous” cell population (cells that has been classified differently in different classification rounds), which we considered as a type of a quality control measure and an indicator aimed at avoiding data overfitting. The occurrence of this type of cells is expected when working with IMC data due to the inaccuracies in cell segmentation and lateral spillover (41, 42). The amount of the ambiguous cells we detected were relatively low in Lum breast cancer but was significantly higher in TN breast cancer.

When comparing the abundance of main cell classes identified in Lum and in TN breast cancer, we observed a much higher percentage of immune cell population in TN than in Lum breast cancer. This confirms previous findings about high TN breast cancer immunogenicity caused by generally higher aggressiveness of this subtype and due to the higher association with occurrence of BRCA1 and BRCA 2 mutations (43–47). Among the immune cells, there were differences in composition between two breast cancer subtypes compared. The most noticeable difference was an identified higher portion of M2 type macrophages in TN breast cancer when compared with Lum, which has also been confirmed in some of the previous studies (48, 49). We also demonstrated a higher abundance of Ki67 expressing CD4+ T lymphocytes and CD8+ T lymphocytes in TN breast cancer.

While recently published CAF-focused studies performed using IMC platform report detection of various CAF populations (30, 50, 51), in our work we were able to identify a broader spectrum of different CAF subsets. Differently to the panel reported by Tornaas et al. (30), where the authors used higher number of CAF associated markers, we focused our panel on aSMA, FAP, PDGFRa and PDGFRb, which have been proven useful in the previous studies for classification of different CAF populations with demonstrated prognostic values (11, 52). Driven by our previous study (52), we have reasons to believe that subclassifying CAFs by their expression profiles of these four markers holds significant prognostic value and that this classification approach could be complemental to functional CAF classification described in literature (53, 54). In addition to aSMA, FAP, PDGFRa and PDGFRb, we included YAP1 and pSMAD2, as markers indicative of CAF functional states and their specific pathways activation. In more detail, YAP1 (Yes associated protein 1) is a well-known transcription coactivator and a key downstream effector in the Hippo pathway (55). It has been known for its role in conversion of physiological fibroblasts into CAFs and its association with CAF directed cancer progression, EMT, metastasis, stemness and chemoresistance (56–60), as well as matrix stiffening and tumor angiogenesis (61, 62). pSMAD2 is an activated form of SMAD2, that when oligomerized with pSMAD3 and SMAD4 acts as a transcription factor and it is an indicator of canonical activation of TGF-b pathway (63, 64). Activation of TGF-b pathway in tumors is known to be associated with CAF transformation from normal fibroblasts and their rapid proliferation, as well as cancer progression and therapy resistance (63, 64). Activation of TGF-b pathway in tumor stroma is known to increase production of collagen rich extracellular matrix, to induce expression of FAP and aSMA, to mediate crosstalk between CAFs and cancer cells and therefore support CAF governing of stemness, EMT, proliferation, metastasis and chemoresistance, and is generally indicative of bad prognosis (65–70). Although our panel had a smaller number of CAF associated markers in comparison with the panel reported by Tornaas et al., (30) focusing on 6 CAF markers allowed implementation of more extensive set of immune and cancer cell markers, enabling more thorough CAF niche profiling.

## 5 Conclusions

In this study, we presented a 42-marker panel, established and validated using thorough quantification methods and a variety of different control tissues. The final panel contained 6 CAF-associated markers (aSMA, FAP, PDGFRa, PDGFRb, YAP1 and pSMAD2). To demonstrate the utility of the panel in identifying diverse cell populations and its potential for detailed profiling of different CAF, immune cell, and cancer cell phenotypes, we applied highly supervised cell classification approach using a modified CELESTA pipeline on IMC data from stained breast cancer tissues. By using the presented IMC panel and adapted cell classification pipeline, we were able to distinguish between 19 different CAF phenotypes, 16 different immune cell phenotypes, and 12 different cancer cell phenotypes.

## Data availability statement

The datasets presented in this study can be found in online repositories. The names of the repository/repositories and accession number(s) can be found below: https://osf.io/eu8ct/. Additional data available upon request.

## Ethics statement

The study was performed in line with guidelines and regulations of the University of Bergen and in accordance with the Declaration of Helsinki Principles. Written informed consent was obtained from all individual participants included in the study.

## Author contributions

HR: Data curation, Formal analysis, Writing – original draft. KF: Resources, Writing – review & editing. IW: Methodology, Writing – review & editing. LA: Resources, Writing – review & editing. AÖ: Funding acquisition, Supervision, Writing – review & editing, Conceptualization, Project administration, Resources. VM: Conceptualization, Data curation, Formal analysis, Methodology, Supervision, Writing – original draft.
