# Supplementary material for: Development of 42 marker panel for in-depth study of cancer associated fibroblast niches in breast cancer using imaging mass cytometry
Source: Front Immunol. 2024 Apr 22;15:1325191. doi: 10.3389/fimmu.2024.1325191 (PMC11070582; doi:10.3389/fimmu.2024.1325191)
Supplement: Supplementary file 1 [file DataSheet_1.docx]

Supplementary material

Development of 42 marker panel for in-depth study of cancer associated fibroblast niches in breast cancer using imaging mass cytometry

Hanna Røgenes1, Kenneth Finne1, Ingeborg Winge1, Lars Akslen1,2, Arne Östman1,3, Vladan Milosevic1*

1Centre for Cancer Biomarkers CCBIO, Department of Clinical Medicine, University of Bergen, Bergen, Norway

2Department of Pathology, Haukeland University Hospital, Bergen, Norway

3Department of Oncology and Pathology, Karolinska Institutet, Solna, Sweden

*Correspondence to: Vladan Milosevic (v.milosevic@uib.no), ORCID: 0000-0001-6991-6795


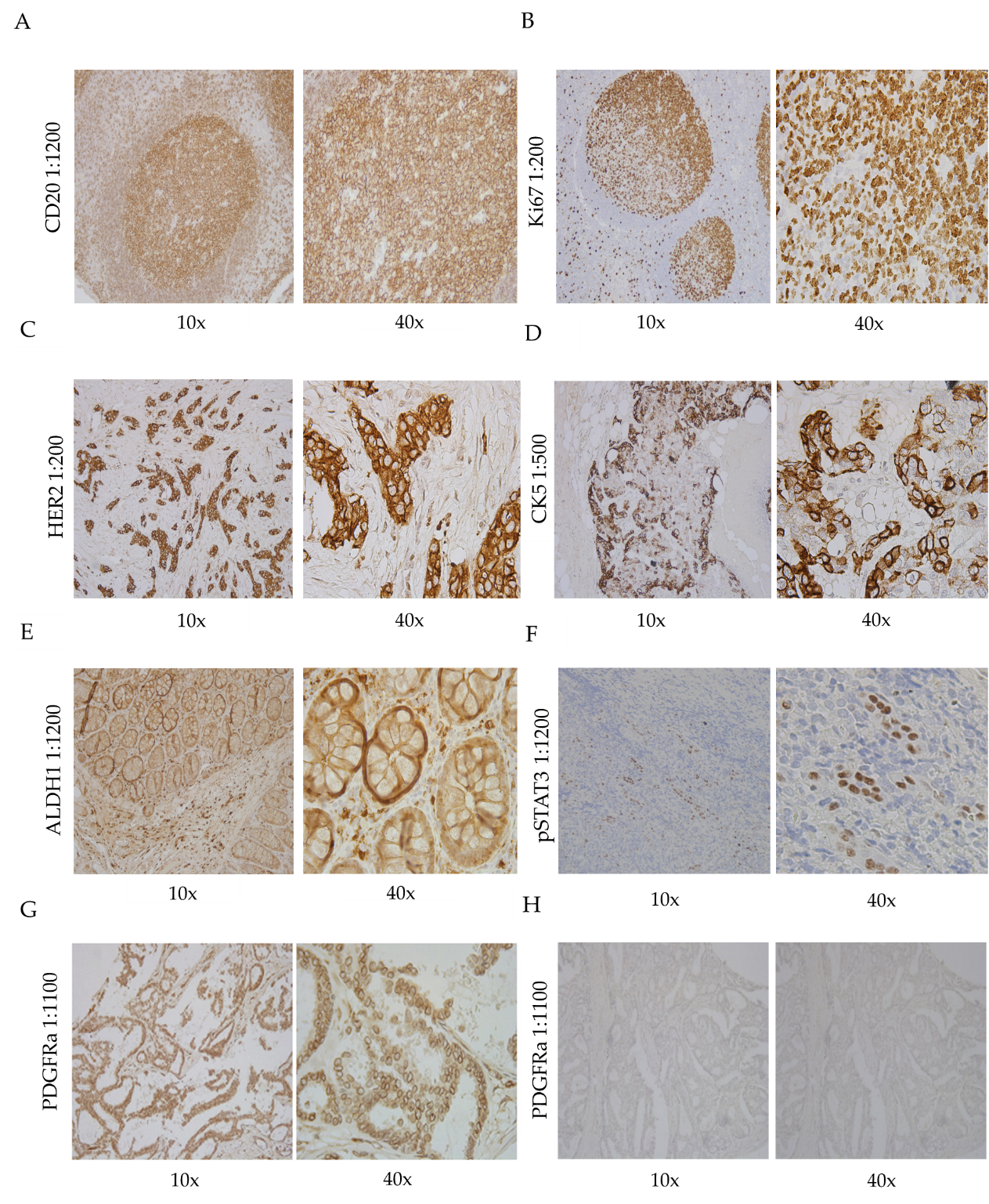


Supplementary Figure 1. Examples of IHC antibody testing. A-E) IHC testing of antibodies conjugated “in-the-house” and antibodies obtained from Standard BioTools as pre-conjugated. F) IHC staining of conjugated pSTAT3 shows good staining quality. G) IHC staining of PDGFRa with unconjugated and H) conjugated antibody.

**
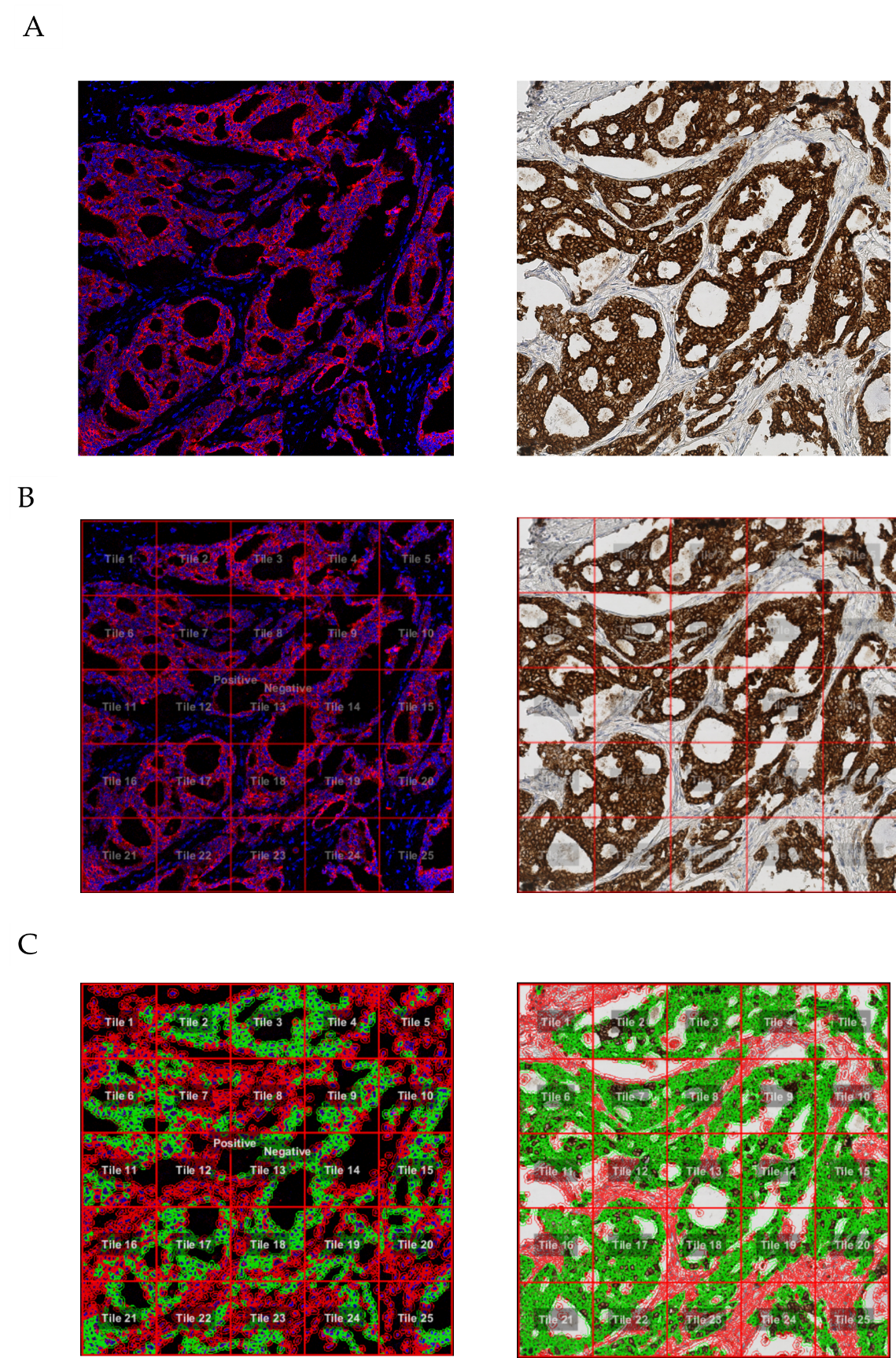
**

Supplementary Figure 2. QuPath-workflow depiction**.** A) Visual representation of both IMC and IHC image of the same core from the consecutive tissue sections as imported into the software. B) Both images are segmented into 25 tiles (visualized as red squares). C) Mean signal intensity and number of detected cell positive for a given marker were calculated per each tile for both images and then compared.


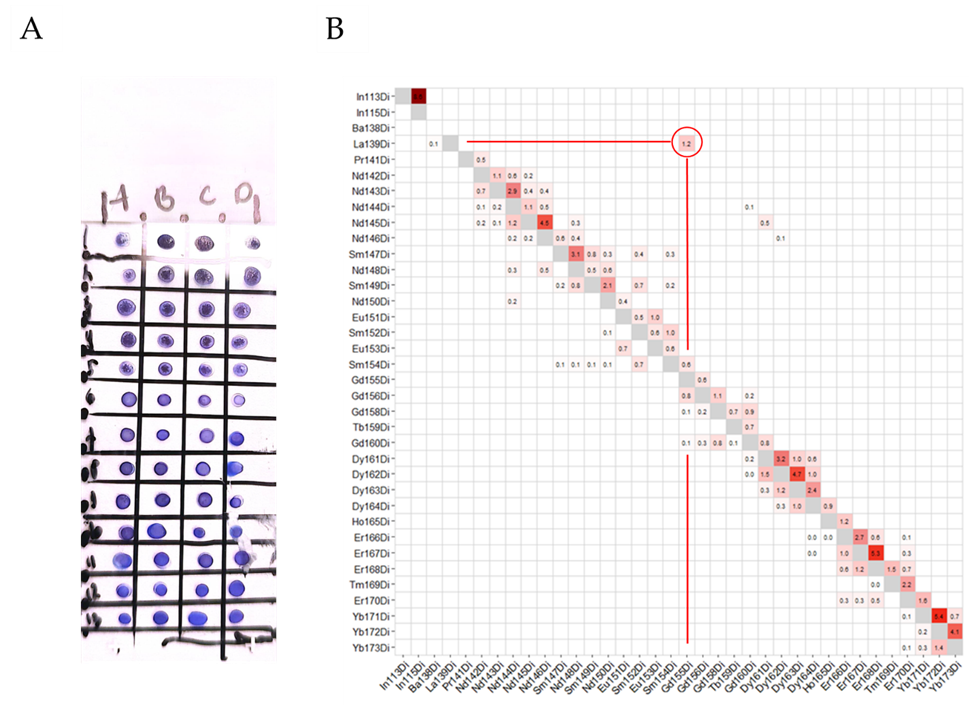


Supplementary Figure 3. Channel crosstalk estimation and correction. A) Agarose slide prepared for channel crosstalk estimation. Each blue dot on the slide represents a drop of 0.4% trypan blue solution together with a unique antibody from the panel. B) Spillover matrix showing the percentage of signal spillover for given channel pairs. In most cases crosstalk has been detected in adjacent channels, indicating the impurities of the metal isotopes used in conjugation. In other cases, e.g. for channel 139La (indicated in red), signal spillover was detected in channel 155Gd (16 Da of difference), possibly caused by oxidation.

Supplementary Figure 4. Signal intensity and the patterns of the “1^st^ level markers” used for the classification of the main cell classes together with the spatial representation of identified main classes.

Supplementary Figure 5. Signal intensity and the patterns of the markers used for the classification of the CAF phenotypes together with the spatial representation of identified CAF phenotypes.

Supplementary Figure 6. Signal intensity and the patterns of the markers used for the classification of the immune phenotypes together with the spatial representation of identified immune phenotypes.

Supplementary Figure 7. Signal intensity and the patterns of the markers used for the classification of the cancer phenotypes together with the spatial representation of identified cancer phenotypes.


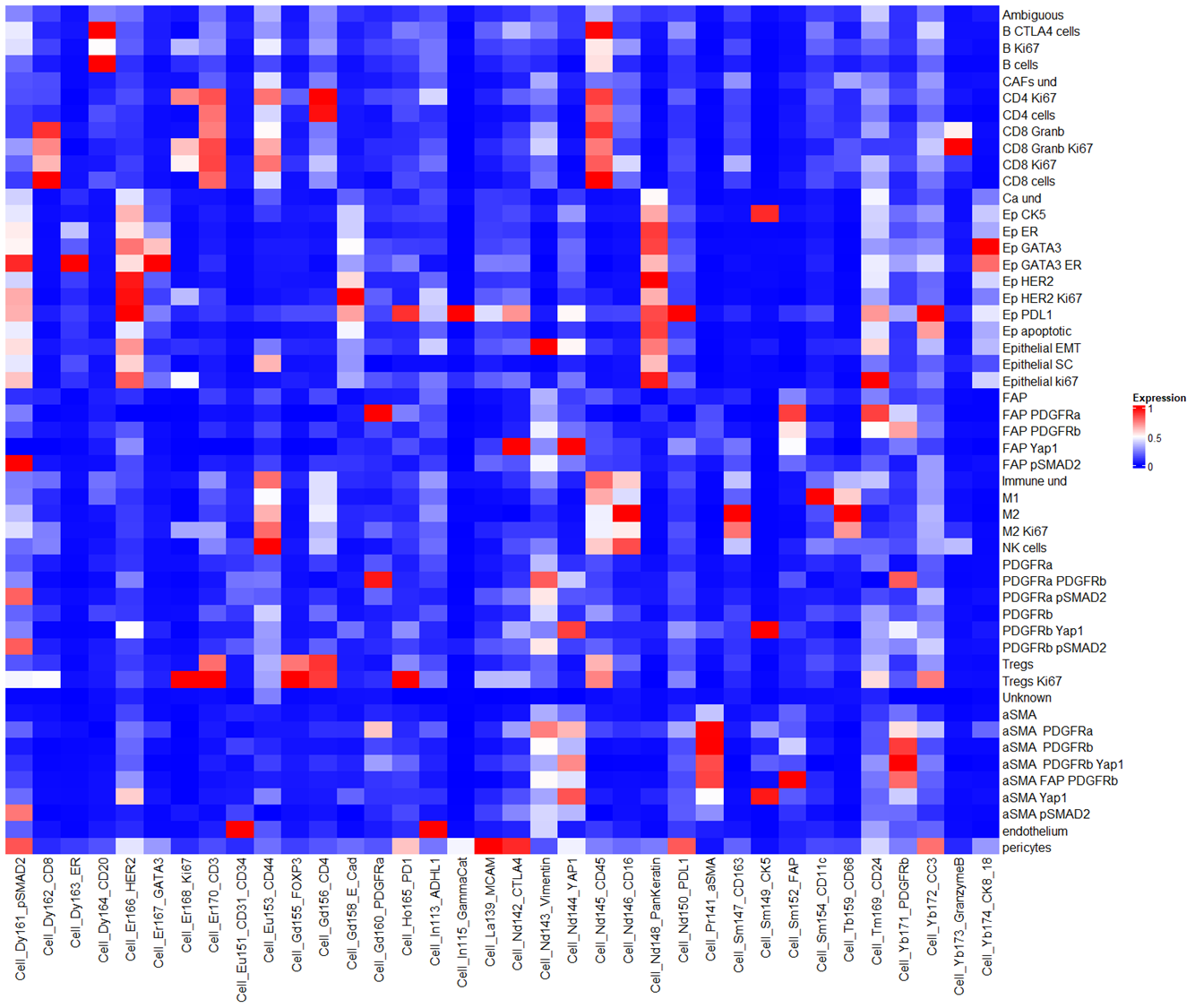


Supplementary Figure 8. Marker expression patterns of identified cell subclasses. Data was subjected to Min-Max normalization with the minimum expression values for each marker mapped to blue, the maximum values to red, and intermediate values to white and shades in between.


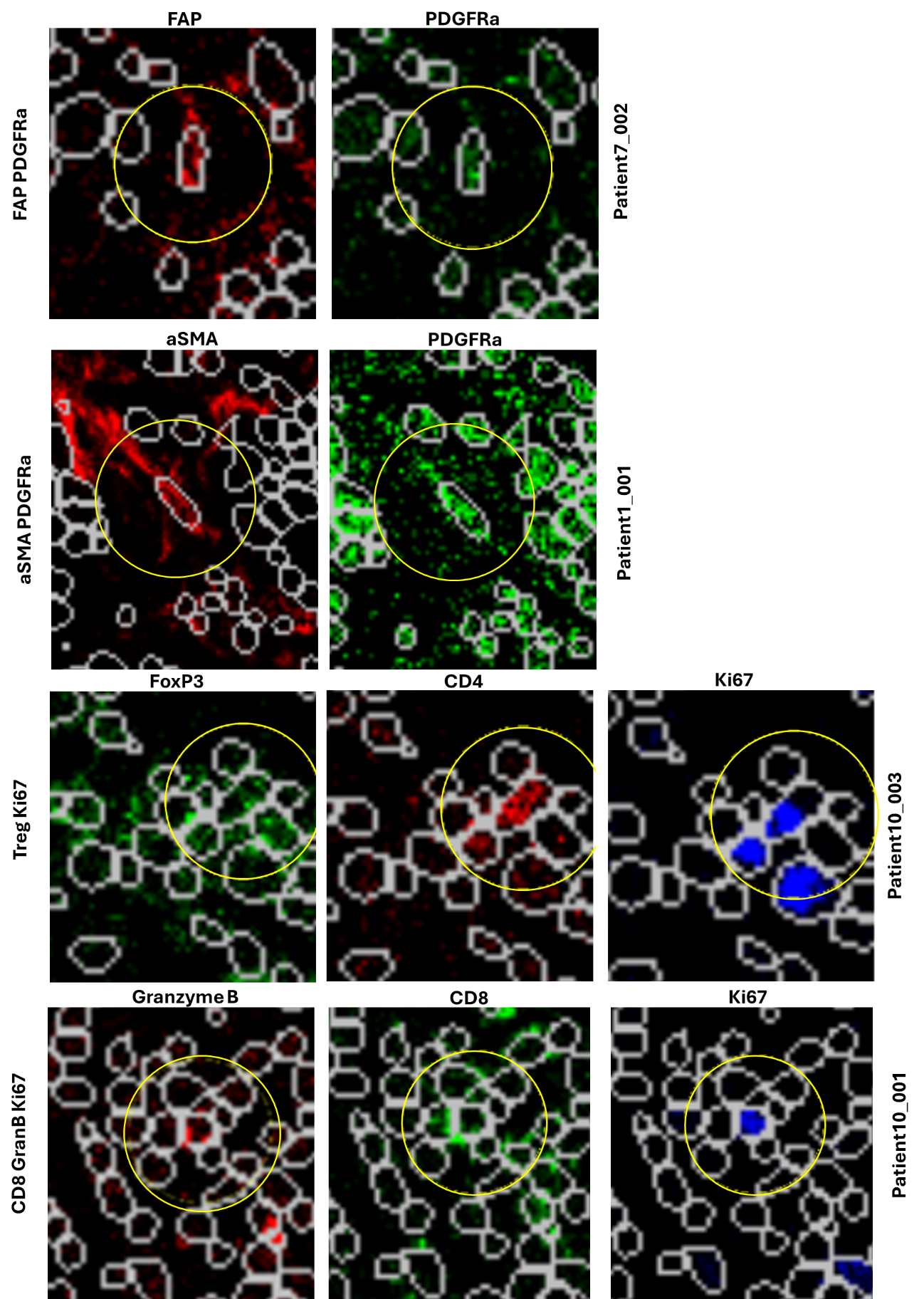


Supplementary Figure 9. Examples of identified rare cell populations, each with distinct marker expression patterns confirming their identity. The cells of interest are indicated by a yellow circle.

Supplementary Figure 10. Abundance of different immune cell phenotypes. A) Relative number of main immune phenotypes, B) relative number of T cell phenotypes, C) relative number of B cell phenotypes, and D) relative number of macrophage phenotypes.

Supplementary Figure 11. Representation of TLSs detected in certain cases of the “pilot-TMA” using CELESTA.

Supplementary Table 1. Summary of different control tissue types from "test-TMA". Each tissue type is presented with an available number of cores and corresponding markers that have been tested. Abbreviations: BC-breast cancer; ER-estrogen receptor; CK-cytokeratin.

| **Tissue type** | **Number of cores** | **Markers** |
| --- | --- | --- |
| ER-positive BC | 2 | ER, CK 8/18, Gamma-catenin and ALDH1 |
| Triple-negative BC | 2 | CK5, CD44, pSMAD2 |
| HER2-enriched BC | 2 | HER2, E-cadherin |
| PDGFRa+ BC | 2 | GATA3, PDGFRa, PanCK |
| Polyp 1 | 2 | FAP, aSMA, Yap1 |
| Placenta | 1 | MCAM, PDL-1 |
| Polyp 2 | 2 | PDGFRb, Vimentin, CD31/34 |
| Tonsil | 3 | Ki67, CD3, CD8, CD20, PD1, CD45, CTLA-4 |
| Lymph node | 3 | FoxP3, CD4, CD11c, granzyme B |
| Lymph node-metastasis | 3 | Cleaved Caspase 3, CD24, CD163, CD68, CD16 |

Supplementary Table 2. Summary of breast cancer tissue types available from "pilot-TMA". The table includes case and core numbering. Abbreviations: BC-breast cancer

| **BC Tissue type** | **Case number** | **Core name** |
| --- | --- | --- |
| Luminal BC | Case  Patient1,  Patient3,  Patient7,  Patient9 | P1: 001-003  P3: 001-003  P7: 001-003  P9: 001-003 |
| Triple-negative BC | Case  Patient2,  Patient4,  Patient6,  Patient8,  Patient10 | P2: 001-003  P4: 001-004  P6: 001-002  P8: 001-003  P10: 001-003 |
| HER2-enriched BC | Case  Patient5 | P5: 001-003 |

Supplementary Table 3. The complete 43 marker panel for imaging mass cytometry on FFPE tissue. Abbreviations: SBT- Standard BioTools; CST- Cell Signaling Technology.

| **Target** | **Metal tag** | **Clone** | **Species/**  **Isotype** | **Supplier** | **Catalog /(lot)** | **Dilution** |
| --- | --- | --- | --- | --- | --- | --- |
| ALDH1 | 113In | 44/ALDH | Mouse, IgG1 | Bd Bioscience | 624084 | 1:100 |
| Gamma-catenin | 115In | 15F11 | Mouse, IgG1, κ | Biolegend | 946402 | 1:1000 |
| MCAM | 139La | Polyclonal | Rabbit, IgG | Atlas Antibodies | HPA008848 (000025781) | 1:200 |
| aSMA | 141Pr | IA4 | Mouse, IgG2a | SBT | 3141017D (2109125-07) | 1:400 |
| CTLA-4 | 142Nd | OTI1G10 | Mouse, IgG1 | Origene | TA810299 (CF810299) | 1:25 |
| Vimentin | 143Nd | D21H3 | Rabbit, IgG | SBT | 3143027D | 1:400 |
| YAP1 | 144Nd | D8H1X | Rabbit, IgG | CST | 14074  (3) | 1:100 |
| CD45 | 145Nd | D9M81 | Rabbit, IgG | SBT | 91H009145 (2210691-28) | 1:100 |
| CD16 | 146Nd | EPR16784 | Rabbit, IgG | SBT | 3146020D (2201609-12) | 1:100 |
| CD163 | 147Sm | EDHu-1 | Mouse, IgG1 | SBT | 3147021D (1622015) | 1:100 |
| PanCk | 148Nd | AE-1/AE-3 | Mouse, IgG1 | SBT | 3148022D (2105105-25) | 1:800 |
| CK 5 | 149Sm | E2T4B | Rabbit, IgG | CST | 71536BF (2) | 1:300 |
| PD-L1 | 150Nd | E1L3N | Rabbit, IgG | SBT | 3150031D (2105065-18) | 1:20 |
| CD31 | 151Eu | EPR3094 | Rabbit, IgG | SBT | (3151025D) 2101803-28 | 1:100 |
| CD34 | 151Eu | ICO115 | Mouse, IgG1 | CST | 41101 (1) | 1:200 |
| FAP | 152Sm | E1V9V | Rabbit, IgG | CST | 66562  (2) | 1:100 |
| CD44 | 153Eu | IM7 | Rat, IgG2b, k | SBT | 3153029D | 1:50 |
| CD11c | 154Sm | EP1347Y | Rabbit, IgG | SBT | 3999999-5 (2109357-28) | 1:100 |
| FoxP3 | 155Gd | PCH101 | Rat, IgG2a, k | SBT | 3155018D (2207305-05) | 1:50 |
| CD4 | 156Gd | EPR6855 | Rabbit, IgG | SBT | 3156033D (0082002) | 1:50 |
| E-cadherin | 158Gd | 24E10 | Rabbit, IgG | SBT | 3158029D | 1:200 |
| CD68 | 159Tb | KP1 | Mouse, IgG1 | SBT | 3159035D (2106189-08) | 1:100 |
| PDGFRa | 160Gd | D1E1E | Rabbit, IgG | CST | 3174T | 1:100 |
| Goat anti-rabbit IgG (H+L) |  | Polyclonal | Goat, IgG | Invitrogen | A16098 | 1:800 |
| pSMAD2 | 161Dy | 138D4 | Rabbit, IgG | CST | 3108BF (11) | 1:50 |
| CD8(a) | 162Dy | C8/144b | Mouse, IgG1 | SBT | 3162034D (2107505-21) | 1:100 |
| ER | 163Dy | D6R2W | Rabbit, IgG | CST | 13258BF/ (2) | 1:100 |
| CD20 | 164Dy | H1 | Mouse, IgG2a, k | SBT | 91H013164 (2208953-29) | 1:500 |
| PD1 | 165Ho | EPR4877(2) | Rabbit, IgG | SBT | 3165039D (2205763-30) | 1:20 |
| HER2 | 166Er | D8F12 | Rabbit, IgG | CST | 4290BF/ (7) | 1:150 |
| GATA3 | 167Er | D13C9 | Rabbit, IgG | CST | 5852BF (2) | 1:50 |
| Ki67 | 168Er | B56 | Mouse, IgG1, κ | SBT | 3168022D | 1:100 |
| CD24 | 169Tm | ML5 | Mouse, IgG1 | Biolegend | 311102  (B280533) | 1:50 |
| CD3 | 170 Er | Polyclonal, C-terminal | Rabbit | SBT | 3170019D (2106278-22) | 1:50 |
| PDGFRb | 171Yb | 28E1 | Rabbit, IgG | CST | 3169BF  (11) | 1:200 |
| Cleaved caspase 3 | 172Yb | 5A1E | Rabbit, IgG | SBT | 3172027D (1192012) | 1:25 |
| Granzyme B | 173Yb | D6E9W | Rabbit, IgG | CST | 79903SF | 1:25 |
| CK 8/18 | 174Yb | C51 | Mouse, IgG1 | SBT | 3174022D (0372007) | 1:100 |
| PanAct | 175Lu | D18C11 | Rabbit, IgG | SBT | 3175032D (3221904) | 1:100 |
| Histone H3 | 176Yb | D1H2 | Rabbit, IgG | SBT | 3176023D  (2107478-20) | 1:500 |
| IMC cell segmentation kit 1/3 | 195Pt | NA | NA | SBT | TIS-00001 (2209049-06) | 1:100 |
| IMC cell segmentation kit 2/3 | 196Pt | NA | NA | SBT | TIS-00001 (2209050-06) | 1:100 |
| IMC cell segmentation kit 3/3 | 198Pt | NA | NA | SBT | TIS-00001 (2209051-06) | 1:50 |
| pSTAT3 | 209Bi | D3A7 | Rabbit, IgG | CST | 9145BF (39) | 1:50 |

Supplementary Table 4. Overview of the 11 markers (obtained from a slide consecutive to IMC slide section) with corresponding Spearman correlation coefficient (R) and p-value for both mean signal intensity and number of positive cells detected. Antibodies are sorted by the strength of correlation in regard to the mean signal intensity, with strong correlation (R >0,7) highlighted in blue, moderate correlation (0,39 < R < 0,7) highlighted in light green, and weak correlation (R < 0,39) highlighted in yellow.

|  | **Mean signal intensity** | | **Number of positive cells detection** | |
| --- | --- | --- | --- | --- |
| **Marker** | **R (spearman correlation)** | **p-value** | **R (spearman correlation)** | **p-value** |
| CD24 | 0.108 | 0.606 | -0.146 | 0.485 |
| CD163 | 0.174 | 0.406 | -0.079 | 0.709 |
| PDL-1 | 0.337 | 0.099 | 0.39 | 0.054 |
| CD44 | 0.547 | 0.005 | 0.294 | 0.154 |
| GranzymeB | 0.878 | <0.001 | 0.889 | <0.001 |
| Ki67 | 0.711 | <0.001 | 0.685 | <0.001 |
| PanCK | 0.595 | <0.001 | 0.501 | 0.011 |
| Vimentin | 0.68 | <0.001 | 0.691 | <0.001 |
| CD8 | 0.75 | <0.001 | 0.89 | <0.001 |
| CD11c | 0.587 | <0.001 | 0.504 | 0.01 |
| CD20 | 0.709 | <0.001 | 0.556 | 0.004 |

Supplementary Table 5. Abundance of “first-level” cell classes per subtype

| Cell Type | Lum | | TN | |
| --- | --- | --- | --- | --- |
|  | N | % | N | % |
| Ambiguous | 5021 | 8.7 | 16335 | 23.7 |
| Endothelium | 1082 | 1.9 | 1165 | 1.7 |
| Pericytes | 212 | 0.4 | 388 | 0.6 |
| Immune cells | 1097 | 1.9 | 8374 | 12.1 |
| Tumor cells | 31996 | 55.5 | 19402 | 28.1 |
| CAFs | 17565 | 30.5 | 21724 | 31.5 |
| Unknown | 689 | 1.2 | 1607 | 2.3 |
| **Σ** | 57662 | 100 | 68995 | 100 |

| Case | Cell Classes | Chi-Square Value | Degree of Freedom | p-value |
| --- | --- | --- | --- | --- |
| Patient 1 | Main cell classes | 6.8 | 12 | 0.87 |
|  | CAFs | 43.3 | 26 | 0.02 |
|  | Immune cells | 77.3 | 20 | 1.1e-08 |
|  | Tumor cells | 20.4 | 20 | 0.43 |
| Patient 2 | Main cell classes | 16.95 | 12 | 0.15 |
|  | CAFs | 113.2 | 24 | 1.57e-13 |
|  | Immune cells | 75.4 | 26 | 1.04e-06 |
|  | Tumor cells | 143.2 | 18 | < 2.2e-16 |
| Patient 3 | Main cell classes | 9.72 | 12 | 0.64 |
|  | CAFs | 96.6 | 22 | 2.6e-11 |
|  | Immune cells | 100.5 | 16 | 2.8e-14 |
|  | Tumor cells | 57.99 | 20 | 1.5e-05 |
| Patient 4 | Main cell classes | 165.7 | 18 | <2.2e-16 |
|  | CAFs | 84. 2 | 18 | 1.56e-10 |
|  | Immune cells | 101.03 | 45 | 3.5e-06 |
|  | Tumor cells | 195.01 | 18 | < 2.2e-16 |
| Patient 6 | Main cell classes | 14.5 | 6 | 0.024 |
|  | CAFs | 10.9 | 6 | 0.09 |
|  | Immune cells | 10.2 | 8 | 0.25 |
|  | Tumor cells | 3.6 | 7 | 0.82 |
| Patient 7 | Main cell classes | 48.5 | 12 | 2.6e-06 |
|  | CAFs | 75.86 | 28 | 2.7e-06 |
|  | Immune cells | 112.9 | 18 | 8.9e-16 |
|  | Tumor cells | 90.9 | 22 | 2.5e-10 |
| Patient 8 | Main cell classes | 50.8 | 12 | 9.9e-07 |
|  | CAFs | 34.3 | 16 | 0.005 |
|  | Immune cells | 155.01 | 18 | <2.2e-16 |
|  | Tumor cells | 47.5 | 18 | 0.0002 |
| Patient 9 | Main cell classes | 1.77 | 12 | 0.9997 |
|  | CAFs | 64.4 | 30 | 0.0003 |
|  | Immune cells | 36.1 | 20 | 0.015 |
|  | Tumor cells | 16.9 | 20 | 0.66 |
| Patient 10 | Main cell classes | 11.78 | 12 | 0.46 |
|  | CAFs | 30.6 | 24 | 0.17 |
|  | Immune cells | 10.32 | 30 | 0.9997 |
|  | Tumor cells | 39.3 | 20 | 0.006 |

Supplementary Table 6. Statistical analysis of intra-case heterogeneity of different cell classes

Supplementary Table 7. Abundance of CAF classes per subtype

| Cell Type | Lum | | TN | |
| --- | --- | --- | --- | --- |
|  | N | % | N | % |
| FAP | 2476 | 14.1 | 3947 | 18.2 |
| FAP PDGFRa | 4 | 0.02 | 0 | 0 |
| FAP PDGFRb | 30 | 0.2 | 21 | 0.1 |
| FAP Yap1 | 3 | 0.02 | 2 | 0.01 |
| FAP pSMAD2 | 4 | 0.02 | 8 | 0.04 |
| PDGFRa | 2395 | 13.6 | 1694 | 7.8 |
| PDGFRa PDGFRb | 5 | 0.03 | 1 | 0.005 |
| PDGFRa pSMAD2 | 137 | 0.8 | 16 | 0.07 |
| PDGFRb | 5573 | 31.7 | 12416 | 57.2 |
| PDGFRb Yap1 | 14 | 0.08 | 2 | 0.01 |
| PDGFRb pSMAD2 | 156 | 0.9 | 67 | 0.3 |
| aSMA | 4701 | 26.8 | 945 | 4.4 |
| aSMA PDGFRa | 5 | 0.03 | 0 | 0 |
| aSMA PDGFRb | 140 | 0.8 | 12 | 0.06 |
| aSMA PDGFRb Yap1 | 2 | 0.01 | 0 | 0 |
| aSMA FAP PDGFRb | 134 | 0.8 | 3 | 0.01 |
| aSMA Yap1 | 53 | 0.3 | 1 | 0.005 |
| aSMA pSMAD2 | 7 | 0.04 | 0 | 0 |
| CAFs und | 1726 | 9.8 | 2589 | 11.9 |
| **Σ** | 17565 | 100 | 21724 | 100 |

Supplementary Table 8. Abundance of immune cell classes per subtype

| Cell Type | Lum | | TN | |
| --- | --- | --- | --- | --- |
|  | N | % | N | % |
| B CTLA4 cells | 6 | 0.6 | 30 | 0.4 |
| B Ki67 | 8 | 0.7 | 63 | 0.8 |
| B cells | 125 | 11.4 | 678 | 8.1 |
| CD4 Ki67 | 0 | 0 | 18 | 0.2 |
| CD4 cells | 219 | 19.96 | 1746 | 20.8 |
| CD8 Granb | 6 | 0.6 | 24 | 0.3 |
| CD8 Granb Ki67 | 0 | 0 | 3 | 0.04 |
| CD8 Ki67 | 0 | 0 | 21 | 0.3 |
| CD8 cells | 247 | 22.5 | 1337 | 15.97 |
| M1 | 96 | 8.8 | 291 | 3.5 |
| M2 | 70 | 6.4 | 2240 | 26.8 |
| M2 Ki67 | 0 | 0 | 6 | 0.07 |
| NK cells | 9 | 0.8 | 23 | 0.3 |
| Tregs | 40 | 3.7 | 101 | 1.2 |
| Tregs Ki67 | 0 | 0 | 2 | 0.02 |
| Immune und | 271 | 24.7 | 1791 | 21.4 |
| **Σ** | 1097 | 100 | 8374 | 100 |

Supplementary Table 9. Abundance of cancer cell classes per subtype

| Cell Type | Lum | | TN | |
| --- | --- | --- | --- | --- |
|  | N | % | N | % |
| Ep CK5 | 144 | 0.5 | 146 | 0.8 |
| Ep ER | 3110 | 9.7 | 161 | 0.8 |
| Ep GATA3 | 14554 | 45.5 | 1929 | 9.9 |
| Ep GATA3 ER | 8 | 0.03 | 0 | 0 |
| Ep HER2 | 5308 | 16.6 | 3415 | 17.6 |
| Ep HER2 Ki67 | 1 | 0.003 | 3 | 0.02 |
| Ep PDL1 | 21 | 0.07 | 13 | 0.07 |
| Ep apoptotic | 142 | 0.4 | 136 | 0.7 |
| Epithelial EMT | 6 | 0.02 | 36 | 0.2 |
| Epithelial SC | 614 | 1.9 | 166 | 0.9 |
| Epithelial ki67 | 59 | 0.2 | 69 | 0.4 |
| Ca und | 8029 | 25.1 | 13328 | 68.7 |
| **Σ** | 31996 | 100 | 19402 | 100 |
